# Choosing to take it cool: a pilot study on changes in gestating sows laying area preference when given access to resting areas with floor-cooling during summer heat

**DOI:** 10.1186/s13028-026-00874-5

**Published:** 2026-07-26

**Authors:** Axel Sannö, Cecilia Hagberg, Anna Wallenbeck, Niclas Högberg

**Affiliations:** 1 Department of Clinical Sciences, Swedish University of Agricultural Sciences, PO-Box 7054, 750 07 Uppsala, Sweden; 2 Farm & Animal Health, Kungsängens Gård 302B, 753 23 Uppsala, Sweden; 3 Department of Applied Animal Science and Welfare, Swedish University of Agricultural Sciences, PO-Box 7024, 750 07 Uppsala, Sweden

**Keywords:** Animal welfare, Comfort, Heat stress, Innovation, Loose-housing, Swine, Under-floor cooling

## Abstract

Heat stress is an increasing concern in pig production as ambient temperatures rise due to climate change. Pigs have limited physiological capacity to dissipate heat and rely largely on behavioural strategies to regulate body temperature. Floor cooling has therefore gained interest as a potential method to alleviate heat stress, particularly since pigs spend large proportions of time lying down. The aim of this pilot study was to investigate whether underfloor cooling influenced lying area preference of gestating sows during warm summer conditions. The study was conducted at the Swedish Livestock Research Centre, Lövsta, Uppsala, Sweden, during July 2025. A group pen (160 m²) housing 31 gestating sows included three concrete resting subsections (A–C) with a total area of 27 m². Underfloor cooling infrastructure had been installed in the concrete slab using embedded plastic tubing. During the experimental period, cold water circulation was applied in subsection A (17 °C) and subsection B (12 °C), while subsection C served as an uncooled control. Sow occupancy of the resting areas was recorded by video during 72 h before cooling commenced and during 72 h with cooling in operation (after a 72 h adjustment period). Still images taken at 30-min intervals were used to calculate occupancy (sows/m²). Data were analysed using mixed linear models. Before cooling, average occupancy did not differ between areas (0.24, 0.22 and 0.26 sows/m² in subsections A, B and C respectively). When underfloor cooling was applied, occupancy increased markedly in the cooled areas (0.44 and 0.41 sows/m² in A and B) compared with the uncooled control (0.27 sows/m²) (*P* < 0.001). Outdoor temperature significantly influenced occupancy in subsection A and B during the cooling period (*P* < 0.001), with higher densities observed at higher temperatures. These results indicate that gestating sows preferentially used cooled resting areas when average outdoor temperatures exceeded 20 °C, hence changing their preference for where to rest. Although based on a short pilot study in a single group pen, the findings indicate that underfloor cooling may be a promising strategy to alleviate heat stress however further studies are needed to examine the benefits and the motivations for the sow’s changes in preference of resting area.

## Findings

Loose-housing of gestating sows is now implemented or is on its way to be implemented in large parts of western pig production countries due to increasing societal pressure to change legislation regarding animal welfare [1]. The welfare of the sows in these systems is also gaining increased interest and research focus. Climate change is expected to lead to higher ambient temperatures and more frequent heat waves, which may increase the risk of heat stress in livestock production systems [2, 3]. Pigs of all ages and especially sows have difficulties regulating their body temperature when temperatures rise having to rely on external sources of cooling since they lack the ability to produce sweat for evaporation cooling [4, 5]. During natural conditions wallowing in water or mud is a common tactic for pigs to stay cool [6]. In modern pig barns such possibilities are lacking. Pigs are particularly sensitive to high temperatures due to their limited physiological capacity to dissipate metabolic heat [4]. Heat stress has been associated with reduced welfare and increased mortality in sows during warm periods [5, 7]. Circulating air by fans, showers and slatted floor areas for resting are commonly used to alleviate heat stress in pig housing. Also, floor cooling is gaining interest, especially for finisher pigs [8] but also for sows [9] in order to lower heat stress and improve welfare, and production. Since sows spend a large proportion of their time lying down, conductive heat transfer to a cooler floor surface may provide effective thermal relief during warm conditions [10]. Previous studies have shown that cooled floors can reduce behavioural and physiological indicators of heat stress in sows and gilts [9, 11]. In pregnant sows, stress can have negative consequences for foetal growth and success at farrowing [12]. The aim of this study was to investigate behaviour changes in lying area preference of gestating sows when underfloor cooling was installed during a warm period.

**Fig. 1:**
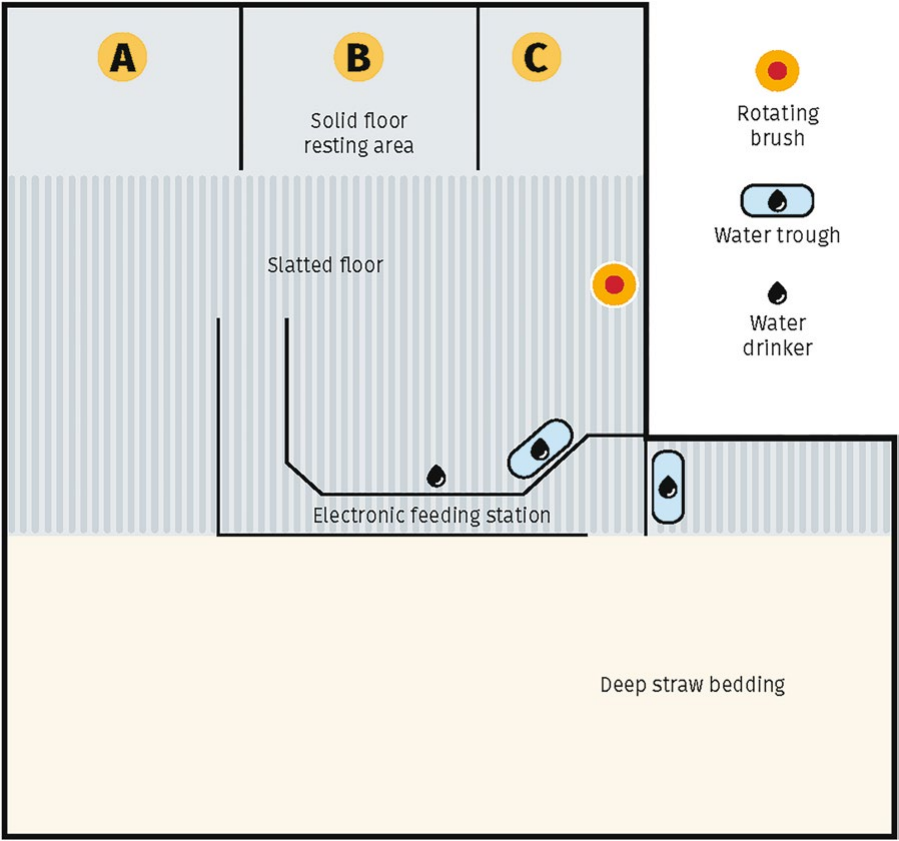
Experimental pen design. Schematic layout and design of the experimental pen with the concrete resting areas denoted with **A**, **B** and **C**, respectively

The study was performed at the Swedish Livestock Research Centre, Lövsta, Uppsala, Sweden from 20 to 28 July 2025. During construction of the gestation unit, plastic tubing (16 mm) with a spacing of 150 mm was cast within the 100-mm concrete slab forming the resting area. A 50-mm insulation layer was installed beneath the concrete slab. During the study period the cooling of the sows’ resting area was commenced by starting to circulate cooled water through the plastic tubing. Hence, the study period consisted of one period without floor cooling, an adjustment period for the sows, and a period with floor cooling. The total area for the experimental group pen was 160 m^2^, with a deep litter bedding area of 68 m^2^ (the bedding was renewed every 8 weeks and fresh barley straw added every other week to maintain a bedding depth of ~ 50 cm), a slatted area of 65 m^2^, and a concrete resting area of 27 m^2^. An electronic feeding station serving pelleted dry feed was placed on the slatted area (Fig. 1). Over the slatted area two shower heads (water flow 1 L min⁻¹) were mounted, each covering an area of ~ 1 m^2^ and activated every 30 min for 60 s between 9 am and 8 pm, year-round. During the study period the group pen housed a dynamic sow group with a total of 31 pregnant sows in various stages of pregnancy. The resting area of the group pen was divided into three subsections A, B and C with an area of 9.6 m^2^, 9.6 m^2^, and 7.9 m^2^ respectively (Fig. 1). The slatted and concrete resting areas were scraped every morning. Straw was provided daily in the resting area (1.5–3 kg day⁻¹). During floor cooling, water was circulated (flow of 0.2m^3^/h) with a set temperature of 17 °C and 12 °C in area A and B, respectively. The water in area C was only circulating without addition of cooled water, hence following the ambient temperature and serving as an uncooled control area. This installation was performed in order to accurately measure temperature of circulating water, however not included in the present study. Evaluation was performed by filming the resting area for 72 h before cooling commenced, followed by 72 h adjustment period before filming again for 72 h when underfloor cooling was in full function. Hourly outdoor temperature data were collected through open-source weather data [13] from three nearby measuring stations, and the values were averaged for the observation periods. Using stills from the films at 30 min intervals, the occupancy on each resting area was calculated by counting resting sows and dividing by the area of the resting area. Data editing, calculation and visualisation of descriptive statistics were performed using Microsoft Excel^®^. Statistical analyses were performed using Statistical Analysis Software (SAS) version 9.4 (SAS Institute, Inc., Cary, NC). The statistical unit of analysis was half hour scan per rest area subsection. Differences in occupancy (number of sows per m^2^) between subsections (A, B, C) were analysed separately for the 72 h period before cooling commenced and 72 h period with underfloor cooling in full function with mixed general linear models using PROC MIXED. Residuals of the continuous outcome variable (occupancy) were examined for normal distribution using PROC UNIVARIATE considering Shapiro- Wilk’s test and normal probability plots and were found to be normally distributed. Results from the statistical analyses are presented as least squares means (LSM) with standard error (± SE). The model included the fixed effect of subsections (A, B, C), the random effect of date and outdoor temperature as a continuous covariate. A P-value < 0.05 was considered significant.

**Fig. 2:**
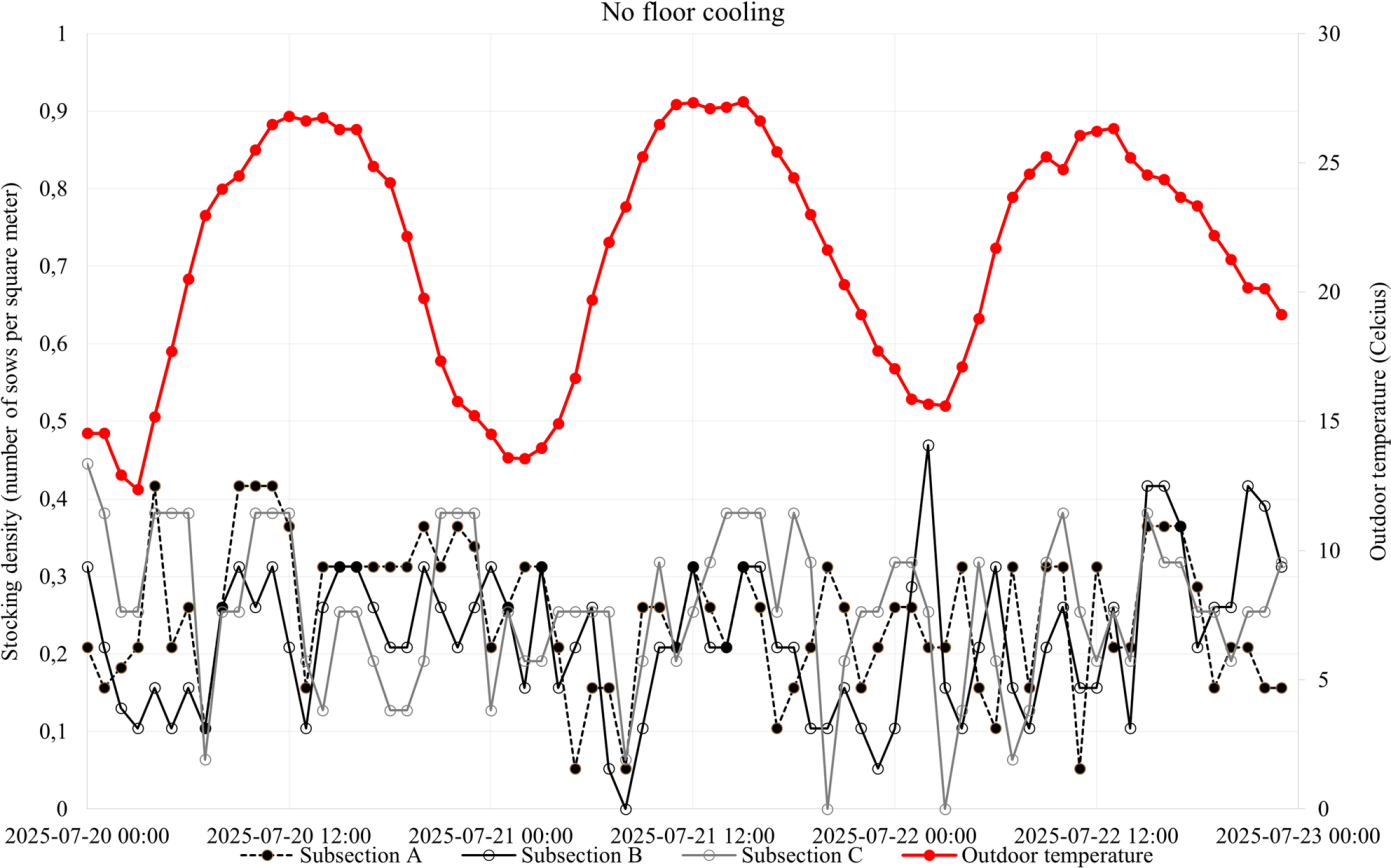
Observations without floor cooling. Time series of occupancy (number of sows per m²) in three subsections (**A**–**C**) of the pen during an observation period without floor cooling (20–23 July 2025). Outdoor temperature (°C) is presented simultaneously (red line, secondary y-axis)

During the first observation period (72 h, 144 observations) without underfloor cooling, the average occupancy was 0.24, 0.22 and 0.26 sows per m² in subsections A, B and C, respectively (Fig. 2). During this period, outdoor temperature ranged from 12.4 °C to 27.3 °C, with an average of 21.5 °C. No significant effect of outdoor temperature on occupancy was detected (*P* = 0.176), but there was a significant effect of subsection (*P* = 0.013). Occupancy was lower in subsection B compared with A and C (0.25^a^ ± 0.016, 0.22^b^ ± 0.016 and 0.26^a^ ± 0.016 sows per m² in subsections A, B and C respectively; LSM ± SE; different superscripts indicate pairwise differences with *P* < 0.05). Thus, the resting areas were broadly comparable in attractiveness to the sows when no thermal differences were present.

**Fig. 3:**
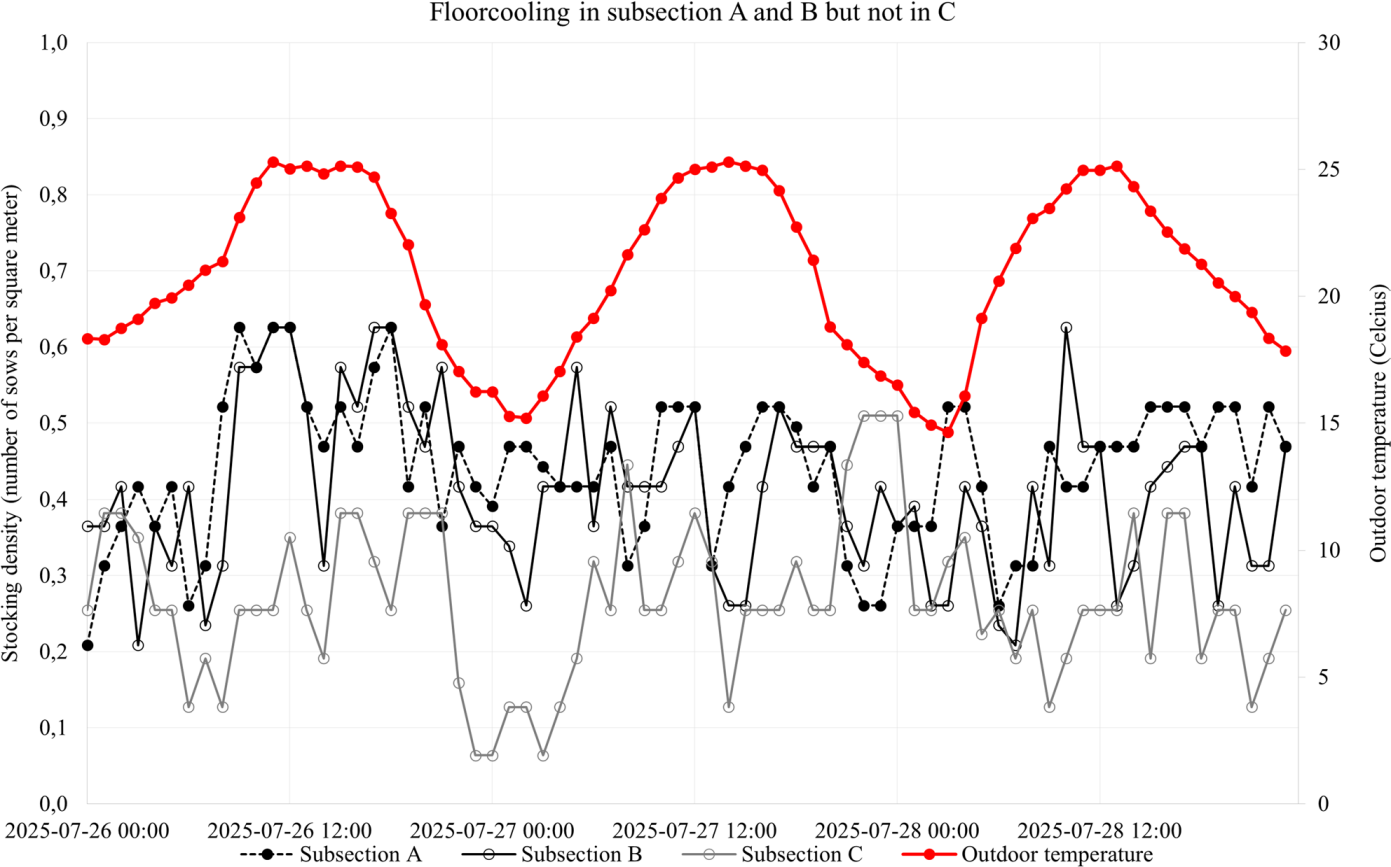
Observations during floor cooling. Time series of occupancy (number of sows per m²) in three subsections (**A**–**C**) during an observation period with floor cooling activated in subsections **A** and **B** (26–28 July 2025), while subsection **C** had no cooling. Outdoor temperature (°C) is shown simultaneously (red line, secondary y-axis)

During the second observation period (72 h, 144 observations), when underfloor cooling was applied in subsections A and B, average occupancy increased markedly in these areas to 0.44 and 0.41 sows per m², respectively, while remaining lower in the uncooled subsection C (0.27 sows per m²) (Fig. 3). Outdoor temperature ranged from 14.6 °C to 25.3 °C, with an average of 20.9 °C. In contrast to the first period, outdoor temperature significantly affected occupancy (*P* < 0.001), with higher occupancy on cooled resting areas observed at higher temperatures. Moreover, occupancy was significantly (*P* < 0.001) higher in the cooled subsections compared with the uncooled control (*P* < 0.001; 0.44^a^ ± 0.012, 0.41^b^ ± 0.012 and 0.27^c^ ± 0.012 sows per m² in subsections A, B and C respectively; LSM ± SE; different superscripts indicate pairwise differences with *P* < 0.05). The occupancy in the uncooled section C was numerically in line with the densities in the first observation period.

These results indicate that on the observation days when temperatures were relatively high, gestating sows preferentially used cooled resting areas during warm conditions, suggesting that conductive floor cooling possibly provided a meaningful thermal benefit. Pigs are known to have limited physiological capacity to dissipate metabolic heat and therefore rely on behavioural strategies to regulate body temperature [4–5]. When opportunities such as wallowing are unavailable in modern housing systems, animals may instead seek cooler surfaces for conductive heat loss [10]. The increased use of cooled resting areas observed in the present study is consistent with this behavioural thermoregulation.

The association between outdoor temperature and occupancy on the cooled resting areas during the cooling period supports this interpretation. As temperatures increased, sows increasingly aggregated in the cooled areas, showing that the cooled floors became more attractive under warmer conditions. Previous studies have shown that floor cooling can reduce behavioural and physiological indicators of heat stress in pigs, including reduced respiration rate and increased lying behaviour [7, 9, 10]. Similarly, improved performance and welfare indicators have been reported in lactating sows provided with cooled floors during warm conditions [14, 15].

Although the present pilot study was conducted over a relatively short period and in one single group pen, the results indicate that underfloor cooling can influence lying area selection by gestating sows. Future studies may include various animal behaviour measurements, including registrations of respiration rates of the sows and more detailed recordings of indoor climate and water temperature. The lack of such recordings represent limitations within the present study, hindering the possibility to detect any possible cooling benefits on the sows.

Since pigs spend large proportions of their time lying down, cooling of resting surfaces may represent an effective strategy to alleviate heat stress in loose-housed systems. Since many modern pig barns, especially in temperate areas such as northern Europe, have underfloor heating already installed to facilitate drying after washing, and heating during winter months, conversion for cooling during summer can easily be achieved. Studies under commercial farm conditions and across longer periods of summer heat are hence needed to evaluate the long-term welfare and production effects of floor cooling for gestating sows.

In conclusion, this pilot study suggests that, given the opportunity, gestating sow will choose to take it cool.

## Data Availability

The datasets used and/or analyzed in this study are available from the corresponding author on reasonable request.

## Declarations

### Prior publication

The data have not been published previously.
